# Role of free radicals and antioxidant status in childhood nephrotic syndrome

**DOI:** 10.4103/0971-4065.78062

**Published:** 2011

**Authors:** S. R. Ghodake, A. N. Suryakar, R. D. Ankush, R. V. Katkam, K. Shaikh, A. V. Katta

**Affiliations:** Department of Biochemistry, PDVVPFs Medical College, Ahmednagar, India; 1 Department of Biochemistry, K. B. N Institute of Medical Sciences, Gulbarga, Karnataka, India; 2 Dr. V. M. Govt. Medical College, Solapur, Maharashtra, India

**Keywords:** Nephrotic syndrome, reactive oxygen species, lipid peroxide, total antioxidant capacity

## Abstract

Nephrotic syndrome (NS) is characterized by heavy proteinuria and hypoalbuminuria. Reactive oxygen species (ROS) seem to play an important role in the etiopathogenesis of proteinuria in NS. This study aims to evaluate the potential role of reactive oxygen species in pathogenesis of NS by estimating the levels of oxidants and antioxidants in children with NS. Thirty patients of NS and thirty age, sex-matched healthy subjects, were selected for the study. As compared to healthy controls, the levels of serum lipid peroxide were significantly elevated while levels of nitric oxide, erythrocyte-superoxide dismutase activity, levels of vitamin C, albumin and total antioxidant capacity were significantly reduced in nephrotic patients. The levels of uric acid and bilirubin were significantly increased in children with NS as compared to controls. There was no significant difference in vitamin E level between patients and controls. It can be concluded that increased ROS generation and decreased antioxidant defense may be related to the pathogenesis of proteinuria in NS.

## Introduction

Nephrotic syndrome (NS) is a common disorder characterized by alteration of permeability of the glomerular capillary wall, resulting in its inability to restrict the urinary loss of proteins, hypoalbuminemia, hyperlipidemia associated with peripheral edema.[1–4]

The exact pathogenesis of excessive protein permeability and other complications of NS remain unknown. An important advance in understanding the pathogenesis of this disease was the observation that ROS are possible mediators of glomerular injury.[5] NS is a consequence of an imbalance between oxidants and antioxidants activity. It was observed that super-oxide mediated oxidative injury degrades the glomerular basement membrane and reduces de novo synthesis of proteoglycans that affects the glomerular permeability.[6–8]

Nitric oxide (NO) is a small signaling molecule that regulates a variety of diverse cellular functions including many physiological and pathological process. This radical has been played a considerable role in the pathogenesis of NS.[9,10]

Super oxide dismutase (SOD) destroys the super oxide radical by converting it to peroxide by dismutation. Vitamin C prevents the pro-oxidant activity of vitamin E by decreasing the activity of α-tocopherol, thereby acting as an antioxidant status and reducing oxidative stress.[11,12] Albumin can react with and neutralize the peroxyl radicals. Uric acid protects not only erythrocytes but also macrophages, by scavenging the singlet oxygen.[13] Bilirubin is an effective antioxidant possibly protecting lipids and lipoproteins against oxidation.[14] Therefore, malondialdehyde (MDA) and NO, indicate the extent of lipid peroxidation caused by reactive oxygen species while SOD, vitamin E, albumin, uric acid bilirubin and total antioxidant capacity determines antioxidant status of body. However, a major unsolved question is whether the elevated glomerular ROS levels are the result of an increased free radical generation alone or are also due to an impaired antioxidant defense.

The present study was planned to evaluate the role of free radicals, oxidant stress and their possible bearing in etio-pathogenesis of NS.

## Materials and Methods

The patients were children admitted with the clinical diagnosis of NS satisfying the International Study of Kidney Diseases in Children (ISKDC) criteria for nephrotic syndrome.[15] A total of 30 consecutive patients were enrolled over a period of one year. All patients with prerenal failure who responded to correction of dehydration and obstructive uropathy, patients with obesity and patients who were on drugs such as vitamins and/or minerals known to alter the oxidative stress parameters were excluded. Thirty age and sex-matched healthy control subjects were selected. Written informed consent was obtained from all subjects. Clearance for the study was obtained from the Ethics Committee of our Institute.

About 10 ml of venous blood was collected, of which 4-5 ml was poured into sterile bulb containing heparin for the estimation of erythrocyte-SOD activity,[16] levels of vitamin E,[17] vitamin C[18] and TAC.[19] Remaining blood was taken into sterile plain bulb for estimation of MDA,[20] NO,[21] albumin,[22] uric acid,[23] bilirubin[24] and cholesterol.[25]

### Statistical analysis

All results were expressed in mean±SD. One way analysis of variance (ANOVA) and Student ‘*t*’ tests was used to compare the results between the patients and the control group. *P* value < 0.05 was considered significant.

## Results

[Table 1] represents the biochemical parameters studied in patients with NS and controls. All results in study group were compared with healthy children.

The levels of MDA were significantly increased in NS children (*P* < 0.001) when compared with controls, whereas serum NO were significantly decreased in NS patients (*P* < 0.001). Total cholesterol levels in NS children were also higher than in the controls (*P* < 0.001). The activity of erythrocyte-SOD, the level of albumin and vitamin C were significantly low (*P* < 0.001) in children with NS as compared to the controls. The concentration of uric acid as well as bilirubin in NS patients was significantly increased. TAC in NS children was also lower than the controls. There was no significant difference in vitamin E levels between patients and controls.

**Table 1:** The levels of serum malondialdehyde, nitric oxide·, RBC-SOD activity, α-tocopherol, ascorbic acid, bilirubin, albumin, cholesterol, uric acid and total antioxidant capacityin controls and patients with nephrotic syndrome

Parameter	Controls	Patients	*P* value
MDA (nmol/ml)	1.53±0.45	3.40±1.38	<0.001
Cholesterol (mg/dl)	170.27±17.67	295.70±58.78	<0.001
Albumin (gm/dl)	3.99±0.52	3.04±60.13	<0.001
Uric acid (mg/dl)	3.44±61.25	4.64±1.32	=0.001
Bilirubin (mg/dl)	0.66±0.10	0.73±0.12	= 0.03
NO• (µmmol/L)	38.80±10.74	25.56±8.78	<0.001
RBC-SOD (U/mg of Hg)	1.94±0.67	1.51±0.81	=0.04
Vitamin E (mg/L)	12.33±2.70	11.90±0.96	=0.1
Vitamin C (mg/dl)	0.70±0.21	0.43±0.27	<0.001
TAC (µmol/L)	832.34±98.12	663.0±62.69	<0.001

## Discussion

Hypoalbuminemia in NS is due to increased glomerular permeability leading to proteinuria. In fact, this molecule may represent the major circulating antioxidant in plasma known to be exposed to continuous oxidative stress.[26] Also, serum albumin is negatively correlated with serum MDA suggesting that hypoalbuminemia leads to the oxidative damage. Our results show significant increase in uric acid levels in study group. This might be due to marked inhibition of renal clearance of uric acid in response to oxidative stress or a consequence of tubular dysfunction or hypertension.[27] Normally, low serum bilirubin concentrations are associated with good health and only high concentrations have any diagnostic significance. The peroxyl radical trapping antioxidant abilities of bile pigments have been reported to be higher than those the serum albumin.[14]

Our finding showed that cholesterol levels were significantly increased in study group. Increase in oxidative damage may be a longer term consequence of sustained hypercholesterolemia.[28,29]

There was a significant elevation in the levels of serum MDA in NS. Since reactive oxygen species can be involved in many degradative processes including lipid peroxidation, and increased generation of reactive oxygen species in glomerular basement membrane. Also, oxidative damage to other molecules along with lipid such as heparan sulfates may lead to increased glomerular permeability.[30,31] A lipid and antioxidant system abnormality play a role in altered glomerular permeability in nephrotic syndrome. In NS, decreased NO end products may result from decreased NO production, increased NO degradation or both. Enhanced inactivation of NO by reactive oxygen species does not seem to be the responsible for a reduced NO bioavailability.[32] In nephrotic children blood pressure is frequently elevated. Therefore, it seems to be possible that hypertension in NS may be associated with impairment in NO system.[33,34]

Lipids are the target molecules for free radicals and this is probably a result of increased consumption of antioxidant components such as erythrocyte-SOD. In the present study reduction in erythrocyte-SOD activity reflects increased susceptibility of RBC membrane to lipid peroxidation.[35]

A significant decrease in plasma ascorbate level was observed in the present study. An some of the other study showed similar results.[36] The regeneration of a-tocopherol involves synergistic reaction between a-tocopherol and ascorbic acid. This recycling reaction leads to formation of dehydroascorbate which is further reduced to ascorbate by a non-enzymatic with reduced glutathione.[37–39] Depletion in ascorbic acid levels were expected in the process of regeneration of a-tocopherol.

Surprisingly, we did not find any differences in vitamin E levels between NS patients and controls. Vitamin E is an important antioxidant that having ability to preserve the intracellular redox balance.[40]

Our study shows that TAC is significantly lowered in NS compared with the levels in healthy children. This is especially expressed in patients with elevated cholesterol suggesting the consumption of some antioxidant components. Low TAC may lead to abnormal lipid peroxidation. TAC was measured before steroid treatment in this study. This is important because glucocorticoids can raise glomerular antioxidant enzyme activity.

In summary, increased free radical generation and decreased antioxidant defenses may be crucial in the pathogenesis of NS. Of considerable interest is the possibility of using this information to develop novel strategies for diagnosis, prognosis and treatment of NS patients are warranted. Further research is needed to explain whether these changes are a cause or consequence of the disease.
